# Patient Satisfaction With Language-Concordant Provider Encounters: A Community-Based Cross-Sectional Survey of Spanish-Speaking Adults

**DOI:** 10.7759/cureus.92447

**Published:** 2025-09-16

**Authors:** Catania Ramos, Megan Hirsch, Jessica Speckart, Shannon Bagot, Alexandra Lopez Vera

**Affiliations:** 1 Medical Education, California University of Science and Medicine, Colton, USA

**Keywords:** clinical communication, health-care equity, language concordance, medical spanish, spanish speaking patients

## Abstract

Background: Hispanic communities in the United States face high rates of chronic disease yet continue to encounter barriers to linguistically concordant care, particularly in underserved regions such as San Bernardino County, California. While professional interpreters improve access, direct provider-patient communication in the patient’s preferred language may yield greater trust, comfort, and satisfaction.

Objective: To assess Spanish-speaking patients’ perceptions of clinical encounters conducted directly in Spanish by their healthcare providers and to compare these experiences to prior interpreter-mediated visits.

Methods: A cross-sectional descriptive study was conducted among Spanish-speaking adults in San Bernardino County who had recently received language-concordant care and had prior experience with interpreter services. Participants were recruited from community centers in Redlands, Colton, and Rialto. A 5-item Likert-scale survey (1 = strongly disagree to 5 = strongly agree) assessed comfort, understanding, emotional support (concerns addressed), trust, and overall satisfaction. Descriptive statistics were calculated.

Results: Fifty participants completed the survey. Mean (standard deviation, or SD) agreement scores for statements comparing encounters with Spanish-speaking providers to participants’ prior interpreter-mediated visits were comfort with provider, 4.68 (0.51); understanding of medical information, 4.64 (0.48); feeling that concerns were addressed (emotional support), 4.50 (0.61); trust in provider, 4.64 (0.56); and overall satisfaction, 4.62 (0.57). Responses clustered at the upper end of the 5-point scale (agree/strongly agree). Comfort had the highest mean score, and the relatively small SDs indicate limited dispersion and consistency across respondents.

Conclusions: Language-concordant care with Spanish-speaking providers was associated with consistently high patient satisfaction across relational and communication domains. These findings support expanding access to bilingual providers and integrating medical Spanish training into healthcare education as strategies to improve trust, comprehension, and engagement in Hispanic communities.

## Introduction

Hispanic communities in the United States face disproportionately high rates of chronic conditions such as diabetes and cardiovascular disease, yet access to linguistically competent care remains a persistent barrier to equitable health outcomes [1,2]. As one of the fastest-growing demographic groups, Hispanic populations frequently experience overlapping challenges, including language barriers, immigration-related stress, socioeconomic constraints, and limited health literacy, which further complicate their ability to access and navigate healthcare services [3].

Despite ongoing legal and institutional efforts to improve access, gaps in language-concordant care, defined as care delivered in the patient’s preferred language by the provider, remain particularly wide in medically underserved regions such as San Bernardino County, California [4]. A growing body of research identifies language discordance between patients and providers as a critical contributor to health disparities, with consequences such as misdiagnoses, reduced adherence to treatment, and delays in care [5,6]. These effects have been linked to higher rates of avoidable hospitalizations [7], poorer control of chronic conditions such as diabetes [5], lower participation in preventive screenings, including mammography and colonoscopy [8], and increased post-discharge complications [5]. Such communication breakdowns are especially harmful in the context of chronic disease management, where sustained engagement and behavioral change are central to effective treatment [9].

Although medical interpreters are vital for bridging communication gaps [10], and their use is federally mandated under Title VI of the Civil Rights Act [11], interpreter-mediated care may not fully replicate the immediacy, emotional resonance, or fluidity of direct, shared-language interactions [12]. Interpreter use can introduce logistical challenges, reduce conversational spontaneity, and limit discussion of sensitive topics such as mental health, reproductive health, or end-of-life preferences [13]. Importantly, research continues to demonstrate the emotional and relational value of language concordance in clinical settings. When providers communicate in a patient’s first language (L1), it can foster deeper emotional connection, increase trust, and enhance patients’ overall perception of care [14]. In Hispanic communities, where values such as personalismo (warmth) and confianza (trust) are culturally central to clinical rapport, language-concordant care is not only linguistically appropriate but also culturally affirming.

Patients who receive care directly in their preferred language are more likely to engage in decision-making, adhere to treatment recommendations, and report high levels of satisfaction with their healthcare experience [15]. These outcomes suggest that expanding access to language-concordant care could meaningfully improve health equity and patient trust.

In regions like San Bernardino County, where Spanish-speaking populations make up a substantial portion of the community and continue to face systemic barriers, understanding how patients experience language-concordant care is essential. This study contributes to the growing evidence base by exploring how Spanish-speaking adults perceive healthcare encounters with Spanish-speaking providers. Findings may inform healthcare training, policy development, and language access strategies aimed at advancing equitable, patient-centered care.

## Materials and methods

Study design

This cross-sectional, descriptive study assessed patient satisfaction among Spanish-speaking adults who had recently received language-concordant medical care in San Bernardino County, California, with data collected from early March through mid-summer 2025. The study focused on patients’ experiences during clinical encounters conducted directly in Spanish by their healthcare providers. All language-concordant providers in this study had verified Spanish proficiency through institutional assessment; however, they were not required to hold formal translation certification or demonstrate dialect matching with participants. The study explored how direct, shared-language communication influenced perceptions of comfort, trust, comprehension, emotional support, and overall satisfaction.

Study setting and participant recruitment

Data were collected in person at community centers located in Redlands, Colton, and Rialto, areas selected for their high concentration of Spanish-speaking residents and cultural relevance. Individuals were approached individually in a private manner during community center programming or events, and invitations to participate were extended one-on-one. Eligibility was determined through participant self-report at the time of recruitment. Bilingual research team members confirmed that participants had recently completed a medical appointment conducted directly in Spanish with a language-concordant provider. Only those who verified this experience were invited to participate in the study. These nonclinical settings provided a neutral and familiar environment, enabling participants to share their healthcare experiences without the pressures often associated with clinical environments. Bilingual members of the research team approached potential participants on-site.

Eligibility criteria

Participants were eligible if they were 18 years of age or older, self-identified as Spanish speakers who required or preferred Spanish-language communication during a recent medical visit, and had just completed a clinical encounter with a Spanish-speaking provider, having also used medical interpreting services in the past. Individuals were excluded if they were younger than 18, declined participation, or did not require any form of language support. Of the individuals approached, two declined participation; no demographic or clinical differences were noted between those who participated and those who declined.

Consent process

Participation was entirely voluntary and anonymous. Before survey administration, team members provided a written informed consent form in Spanish, which outlined the study’s purpose, procedures, risks, and participants’ right to withdraw at any time. No identifiable information (such as names, addresses, phone numbers, or medical record numbers) was collected, and participants were informed that there would be no monetary or other compensation for their involvement.

Survey instrument and data collection

The survey items were designed to assess participants’ perceptions of their language-concordant encounter in relation to prior experiences using medical interpreters. Each statement prompted a comparative reflection, allowing respondents to express preferences based on their broader healthcare experiences.

The study used a brief verbal survey consisting of five statements, each rated on a 5-point Likert scale (1 = strongly disagree to 5 = strongly agree). Items assessed comfort with the provider, understanding of medical information, concerns addressed (emotional support), trust in the provider, and overall satisfaction. The survey items were adapted from prior literature on patient-provider communication and satisfaction among Spanish-speaking patients [16,17]. The instrument was reviewed by two subject matter experts in medical Spanish education and patient-provider communication. A brief pilot with Spanish-speaking adults was conducted to confirm clarity and comprehension, with only minor wording adjustments required.

All surveys were administered individually in a one-on-one format by bilingual research team members to ensure privacy and minimize group influence.

Overall satisfaction

All questions were written in plain, culturally appropriate Spanish to ensure accessibility for participants with diverse literacy levels. Surveys were administered immediately following each clinical encounter with a Spanish-speaking provider. Each survey took approximately five minutes to complete. In total, 50 surveys were collected between early March and mid-summer 2025. All responses were entered into a secure, de-identified database for analysis.

Data analysis

Descriptive statistics were calculated using Microsoft Excel (Microsoft® Corp., Redmond, WA, USA). For each survey item, mean scores and standard deviations (SDs) were computed to identify trends in patient satisfaction based on participants’ comparative reflections. Although Likert-type responses are ordinal, reporting means and SDs is common in medical education research and generally robust when distributions are not severely skewed; therefore, means (SD) are presented for interpretability. Given the limited sample size and the ordinal nature of the data, no inferential statistical tests (e.g., t-tests or chi-square analyses) were performed.

Ethical considerations

The study protocol was reviewed and approved by the California University of Science and Medicine Institutional Review Board (IRB). It was classified as exempt under federal guidelines (45 CFR 46) and was approved under IRB Protocol #: HS-2025-11. The research posed minimal risk, involved no identifiable information, and followed all institutional and federal guidelines for ethical conduct in human subjects research.

## Results

Patient responses indicated consistently high levels of satisfaction across the five assessed items, as summarized in Table 1. Item means ranged from 4.50 to 4.68 on the 5-point scale, with responses concentrated at the upper end of the scale (i.e., agree/strongly agree). “I feel more comfortable communicating with a provider who speaks Spanish in comparison to using a medical interpreter” received the highest rating (M = 4.68, SD = 0.51). “I understand the medical information more clearly when I speak directly with a Spanish-speaking provider instead of an interpreter” (M = 4.64, SD = 0.48) and “I feel more trust in my medical provider when speaking Spanish than when using an interpreter” (M = 4.64, SD = 0.56) were similarly high. “I feel that my concerns are better addressed by a Spanish-speaking provider in comparison to using an interpreter” was slightly lower yet strongly positive (M = 4.50, SD = 0.61). “In general, I am more satisfied with my visit when my provider speaks Spanish when compared to using an interpreter” also reflected high satisfaction (M = 4.62, SD = 0.57). All 50 participants completed the five items; there were no missing data.

**Table 1:** Patient Satisfaction Ratings for Language-Concordant Encounters With Spanish-Speaking Providers (n = 50) Each question’s answers were ranked on a scale from 1 to 5. 1 indicated “totalmente en desacuerdo” or “strongly disagree,” 2 indicated “en desacuerdo” or “disagree,” 3 indicated “neutro” or “neutral,” 4 indicated “de acuerdo” or “agree,” and 5 indicated “totalmente de acuerdo” or “strongly agree.” SD: standard deviation

Question	Mean Score	SD
Question 1: I feel more comfortable communicating with a provider who speaks Spanish in comparison to using a medical interpreter.	4.68	0.51
Question 2: I understand the medical information more clearly when I speak directly with a Spanish-speaking provider instead of an interpreter.	4.64	0.48
Question 3: I feel that my concerns are better addressed by a Spanish-speaking provider in comparison to using an interpreter.	4.50	0.61
Question 4: I feel more trust in my medical provider when speaking Spanish than when using an interpreter.	4.64	0.56
Question 5: In general, I am more satisfied with my visit when my provider speaks Spanish when compared to using an interpreter.	4.62	0.57

Interpretation and patterns

The findings from this study suggest that language-concordant care is associated with consistently positive patient experiences across key dimensions - emotional comfort, communication clarity, perceived support, and interpersonal trust. The high mean scores across all survey items reinforce the notion that direct communication in a patient’s preferred language enhances both the relational and informational quality of clinical encounters.

While this study did not include a comparator group involving interpreter-mediated care, the results align with prior research highlighting the emotional and communicative benefits of language-concordant provider-patient interactions. These findings add to the growing body of evidence advocating for increased access to linguistically aligned care, particularly in regions serving large populations with limited English proficiency.

In San Bernardino County, where a substantial portion of the population identifies as Spanish-speaking and continues to face systemic barriers to healthcare, these results point to the need for broader institutional efforts. Strategies such as incorporating medical Spanish into provider training, recruiting more bilingual clinicians, and promoting culturally responsive communication practices may help reduce linguistic disparities and improve patient engagement in underserved communities.

## Discussion

Principal findings

This study explored Spanish-speaking patients’ perceptions of satisfaction with language-concordant healthcare encounters in San Bernardino County, California. Using a structured survey, we assessed five key domains - comfort, understanding, emotional support, trust, and overall satisfaction - providing quantifiable insight into how patients perceived their care. Participants reported consistently high satisfaction across all domains. The highest-rated domain was comfort with the provider, suggesting that direct language alignment may foster a more relaxed and confident clinical environment. While this study focused on measurable perceptions of satisfaction rather than in-depth qualitative narratives, the findings nonetheless underscore the important role of shared language in enhancing patient experience, especially for populations that have historically encountered linguistic barriers in healthcare settings.

Interpretation in the context of existing literature

These findings align with prior research demonstrating that direct communication between Spanish-speaking patients and Spanish-speaking providers enhances the quality of the patient-provider relationship. For example, Chandrashekar et al. found that patients report greater satisfaction and engagement in their care when their provider speaks Spanish, while Sharkiya identified improved continuity of care under similar circumstances [9,15]. Numerous studies have shown that language concordance contributes to better outcomes, including greater trust, satisfaction, and adherence to treatment recommendations [3-5]. These benefits are especially evident in Hispanic communities, where cultural values such as personalismo and confianza shape patient expectations and interactions [14,15]. These findings indicate that the study successfully met its stated objectives of assessing Spanish-speaking patients’ satisfaction with language-concordant encounters and comparing these experiences to interpreter-mediated care.

While interpreter services remain essential where direct language alignment is not feasible, existing literature [12,13] has documented their limitations, including reduced conversational flow, diminished emotional connection, and discomfort when discussing sensitive topics. Although our study did not include a comparator group using interpreters, the consistently high satisfaction reported in language-concordant encounters reinforces earlier conclusions about the relational and communicative benefits of shared-language care. These results underscore the importance of expanding access to bilingual providers as a strategy to advance equity, trust, and patient-centered care in linguistically diverse populations.

Strengths and limitations

A key strength of this study lies in its community-based, non-clinical data collection settings, which helped reduce social desirability bias by positioning participation outside the perceived authority of healthcare institutions. Additionally, the survey instrument - written in plain, culturally appropriate Spanish - was accessible to individuals with varied literacy levels, contributing to a high response rate with no missing data. Although the instrument relied on closed-ended Likert-scale questions, the use of validated domains such as comfort, understanding, support, trust, and overall satisfaction provided structured insight into participants’ perceptions of language-concordant care.

However, several limitations should be acknowledged. First, the cross-sectional design captures perceptions at a single point in time, limiting the ability to assess long-term outcomes such as treatment adherence, clinical effectiveness, or continuity of care. Second, while the survey offered valuable quantitative measures, its brevity limited the opportunity to capture more nuanced or qualitative dimensions of the patient experience, such as emotional resonance or communication style. Third, the study did not stratify responses based on participants’ prior exposure to interpreter-mediated care or bilingual providers, which may have influenced their satisfaction levels. Fourth, the generalizability of findings is limited to Spanish-speaking patients in San Bernardino County and may not extend to other populations or regions. Finally, while the instrument was pilot-tested for clarity, formal reliability testing was not conducted, and future studies should incorporate reliability and validity assessments. Finally, given the exploratory nature of this study and the modest sample size, descriptive statistics were used to summarize patient responses. While inferential testing was not applied, the descriptive approach aligned with the study’s objectives and provided a foundation for future research with larger samples and validated instruments.

Future research should investigate how patients' prior language experiences shape trust, communication preferences, and satisfaction with care. Additionally, qualitative or mixed-methods approaches conducted shortly after clinical encounters could offer richer insights into the interpersonal and cultural dynamics that shape the effectiveness of language-concordant communication.

## Conclusions

This study underscores the importance of preparing future providers to deliver language-concordant care in regions such as San Bernardino County, where Spanish-speaking populations represent a substantial portion of the community and continue to face systemic barriers to healthcare. Direct Spanish-language communication with providers was associated with greater patient satisfaction across multiple dimensions, highlighting the relational and informational benefits of shared language.

Recognizing Spanish proficiency as a core clinical competency - and pairing it with training in cultural humility and interpersonal communication - can help institutions advance patient-centered care and reduce disparities. These findings have implications for curriculum development and policy initiatives aimed at promoting health equity in linguistically diverse communities. Future research should further examine how language-concordant care impacts long-term outcomes, including adherence, continuity of care, and patient trust, to strengthen the evidence base and guide institutional efforts.
